# Relating drug–protein interaction network with drug side effects

**DOI:** 10.1093/bioinformatics/bts383

**Published:** 2012-09-03

**Authors:** Sayaka Mizutani, Edouard Pauwels, Véronique Stoven, Susumu Goto, Yoshihiro Yamanishi

**Affiliations:** ^1^Bioinformatics Center, Institute for Chemical Research, Kyoto University, Gokasho Uji, Kyoto 611-0011, Japan, ^2^Mines ParisTech, Centre for Computational Biology, 35 rue Saint-Honoré, F-77305 Fontainebleau Cedex, France, ^3^Institut Curie, F-75248, Paris, France, ^4^INSERM U900, F-75248 Paris, France and ^5^Division of System Cohort, Medical Institute of Bioregulation, Kyushu University, 3-1-1 Maidashi, Higashi-ku, Fukuoka, Fukuoka 812-8582, Japan

## Abstract

**Motivation:** Identifying the emergence and underlying mechanisms of drug side effects is a challenging task in the drug development process. This underscores the importance of system–wide approaches for linking different scales of drug actions; namely drug-protein interactions (molecular scale) and side effects (phenotypic scale) toward side effect prediction for uncharacterized drugs.

**Results:** We performed a large-scale analysis to extract correlated sets of targeted proteins and side effects, based on the co-occurrence of drugs in protein-binding profiles and side effect profiles, using sparse canonical correlation analysis. The analysis of 658 drugs with the two profiles for 1368 proteins and 1339 side effects led to the extraction of 80 correlated sets. Enrichment analyses using KEGG and Gene Ontology showed that most of the correlated sets were significantly enriched with proteins that are involved in the same biological pathways, even if their molecular functions are different. This allowed for a biologically relevant interpretation regarding the relationship between drug–targeted proteins and side effects. The extracted side effects can be regarded as possible phenotypic outcomes by drugs targeting the proteins that appear in the same correlated set. The proposed method is expected to be useful for predicting potential side effects of new drug candidate compounds based on their protein-binding profiles.

**Supplementary information:** Datasets and all results are available at http://web.kuicr.kyoto-u.ac.jp/supp/smizutan/target-effect/.

**Availability:** Software is available at the above supplementary website.

**Contact:** yamanishi@bioreg.kyushu-u.ac.jp, or goto@kuicr.kyoto-u.ac.jp

## 1 INTRODUCTION

Predicting and countering the side effects of a new drug during its developmental phase remain important to the drug's overall commercial success. Side effects are responsible for a significant number of cases where premarketed drugs fail during clinical trials. Identifying the underlying mechanisms of side effects is a challenging task, often because of the drugs' pleiotropic effects on a biological system. Most drugs are small compounds that target and interact with proteins to induce perturbations in the proteins network. This underscores the need of system-wide approaches for predicting drug side effects by linking different scales of drug actions; drug–protein interactions (molecular scale) and relationships between drugs and side effects (phenotypic scale) (Fliri *et al*., 2005, 2007; Tatonetti *et al*., 2009).

The most widely used approach to identify possible side effects for a drug is to use its chemical structure information, based on the observation that drug chemical structures can direct the ligand promiscuity toward protein targets (Bender *et al*., 2007). For example, Scheiber *et al*. (2009) investigated correlations between drug chemical substructures and side effects, although they do not provide any prediction frameworks for new drug molecules. Yamanishi *et al*. (2010) proposed a method to predict the pharmacological effects of drugs using their chemical structures. They then inferred drug-target interactions, but their method cannot be directly applied to the prediction of high-dimensional side effect profiles. Atias and Sharan (2011) proposed a method to predict side effects from chemical structure data using canonical correlation analysis (CCA). This work was pioneering in terms of simultaneous prediction of many side effects. Pauwels *et al*. (2011) proposed a method to relate drug chemical fragments with side effects using sparse CCA (SCCA), and used the chemical fragments to predict side effect profiles. However, these chemical structure-based methods cannot provide any biological interpretations regarding the underlying mechanisms at a molecular interaction level.

Chemically unrelated drugs may present similar side effects because they happen to share common off-target proteins (Finlaysona *et al*., 2004). On the basis of this observation, Campillos *et al*. (2008) used side effect similarity of marketed drugs to predict drug pairs with common protein targets. Xie *et al*. (2009) identified drug off-targets by docking the drug into protein binding pockets similar to that of its primary target, followed by mapping the proteins with the best docking scores to known biological pathways, thus predicting potential side effects. Using a similar docking approach, Wallach *et al*. (2010) searched for correlated pairs of side effects and biological pathways. These docking-based methods depend heavily on the availability of protein 3D structures, which presents serious limitations as many useful drug targets are membrane proteins, for which very few structures are available.

From a system-wide viewpoint, Fliri *et al*. (2005) performed a biological spectra-based approach to investigate the correlation between drug-targeted proteins and their side effects. They clustered drugs based on their biological spectra (i.e. their ability to inhibit each of 92 selected proteins) and revealed a correlation between the chemical structures of the corresponding drugs and their biological activity in terms of protein inhibition profile. They further showed that drugs with similar protein inhibition profiles tend to cause similar side effects (Fliri *et al*., 2007). However, it remains difficult to experimentally determine the link between drug-targeted proteins and side effects in a large-scale datasets in a cost-effective and efficient manner (Whitebread *et al*., 2005). Therefore, there is a strong incentive to develop computational approaches for analyzing and predicting drug side effects.

In this article, we examine the correlation between drug–protein interactions and their side effects on a large scale, without limiting ourselves to proteins of known 3D structures. We identify correlated sets of proteins and side effects based on the co-occurrence of drugs in protein-binding profiles and in side effect profiles using SCCA. Results demonstrate that proteins in the same correlated set tend to be involved in only a few biological pathways even if their molecular functions are different. We also address that the side effects in each correlated set present possible outcomes from drug perturbations of corresponding proteins. The originality of the proposed method lies in the integration of drug–protein interactions at a molecular scale and drug side effect relationships at a phenotypic scale. Performance evaluation showed that this method works better than the case where chemical structure profiles are used in the SCCAframework. We also conduct a comprehensive side effect prediction for drug molecules stored in DrugBank without side effect information and confirm interesting predictions using independent source of information.

## 2 MATERIALS

### 2.1 Drug–protein interaction profiles and side effect profiles

Drug–protein interactions were obtained from DrugBank (Wishart *et al*., 2008) and Matador (Günther *et al*., 2008). Both the primary target proteins as well as all proteins known to directly interact with a particular drug were used for analysis. Side effect information was obtained from SIDER, which accumulates reported side effects from package inserts for marketed drugs (Kuhn *et al*., 2010). In total, 658 drugs had both targeted protein and side effect information. This led to the construction of 5074 drug–protein interactions containing 1368 targeted proteins and 49 051 drug side effect pairs containing 1339 side effects. Each of the 658 drugs was represented by a 1368-dimensional protein-binding profile and 1339-dimensional side effect profile, which encodes for the presence or absence of proteins (side effects) by 1 or 0, respectively.

### 2.2 Chemical structures

To encode drug chemical structures, a fingerprint was used, which consisted of 881 chemical substructures defined in the PubChem database (Li *et al*., 2010). This resulted in a binary profile referred to as chemical substructure profile.

### 2.3 Annotation of drug-targeted proteins

265 pathway maps and 546 BRITE terms were obtained from Kyoto Encyclopedia of Genes and Genomes (KEGG) (Kanehisa *et al*., 2010). Note that ‘global pathways’ and ‘disease pathways’ were excluded from the pathway set. Fourteen molecular function categories used in the protein annotations were also obtained from KEGG. 22 043 biological process terms and 9971 molecular function terms were obtained from Gene Ontology (GO) (Ashburner *et al*., 2000).

## 3 METHODS

### 3.1 Ordinary canonical correlation analysis (OCCA)

Suppose that we have a set of *n* drugs with *p* targeted protein features and *q* side effect features. Each drug is represented by a targeted protein feature vector **x**= (*x*_1_,...,*x_p_*)*^T^*, and by a side effect feature vector **y** = (*y*_1_,...,*y_q_*)*^T^*.

We consider two linear combinations for targeted proteins and side effects as *u_i_* = ***α**^T^***x***_i_* and *v_i_* = ***β**^T^***y***_i_* (*i* = 1,2,...,*n*), where ***α*** = (*α*_1_,...,*α_p_*)^*T*^ and ***β*** = (*β*_1_,..., *β_q_*)*^T^* are weight vectors. We attempt to find weight vectors ***α***and ***β***which maximize the following canonical correlation coefficient:
(1)
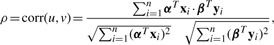

where *u* (respectively, *v*) is centered. *u* (respectively, *v*) is called ‘canonical component’, score.

Let X denote the *n*×*p* matrix defined as *X* = [**x**_1_,...,**x***_n_*]*^T^*, and Y denote the *n*×*q* matrix defined as *Y* = [**y**_1_,...,**y***_n_*]*^T^* .

Then the maximization problem can be written as follows:
(2)
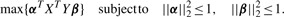


### 3.2 Sparse canonical correlation analysis (SCCA)

Most elements in the weight vectors ***α*** and *****β***** in OCCA are non-zeros, which makes it difficult to interpret the result. In practice, it is desirable to find weight vectors that have large correlation, but that are also sparse for easier interpretation.

To impose the sparsity on ***α*** and *****β*****, we consider the following maximization problem with additional *L*_1_ penalty terms:
(3)
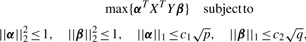

where ||·||_1_ is *L*_1_ norm (the sum of all absolute values of the vector elements), and *c*_1_ and *c*_2_ are parameters to control the sparsity (0 *< c*_1_ ≤ 1 and 0*< c*_2_ ≤ 1). For simplicity, the same value is used for *c*_1_ and *c*_2_ in this study. The CCA with *L*_1_ penalties is referred to as SCCA. The weight vectors *α* and *β* can be optimized by solving penalized matrix decomposition of the matrix *Z* = *X^T^ Y* (Witten *et al*., 2009).

To obtain multiple canonical components, we perform a deflation manipulation iteratively as follows: *Z*^(^*^k^*+^1)^← *Z*^(^*^k^*^)^ − *d_k_****α****_k_* *****β******_k_^T^,* where *Z*^(^*^k^*^)^ is the input of step *k* (*Z*^(1)^ = *X^T^ Y*), ***α****_k_* and *****β******_k_* are the weight vectors, and *d_k_* is singular value obtained in the *k*-th step (corresponding to the *k*-th component) (*k* = 1,2,...,*m*). Finally, we obtain *m* pairs of weight vectors (***α***_1_,*****β*****_1_),...,(***α****_m_*,*****β******_m_*). Proteins and side effects with non-zero weights in the weight vectors are extracted as correlated sets.

### 3.3 Prediction of side effect profiles for new molecules

Given the profile of targeted proteins **x**_new_ for a drug of unknown side effects, we consider predicting its potential side effect profile **y**_new_ based on the weight vectors {***α****_k_*}*_k_*_=__1_*^m^* and {*****β******_k_* }*_k_*_=__1_*^m^*.

We use the following prediction score for a given molecule:
(4)
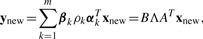

where *A* = [***α***_1_,...,***α****_m_*], *B* = [*****β*****_1_,...,*****β******_m_*] and Λ is the diagonal matrix whose elements are canonical correlation coefficients. If the *j*-th element in **y**_new_ has a high score, the new molecule is predicted to have the *j*-th side effect (*j* = 1,2,...,*q*). The same prediction score was proposed in the previous work (Pauwels *et al*., 2011).

### 3.4 Enrichment analyses of targeted proteins

Let *G_c_* denote the set of extracted proteins in component *c* and *G* denote the set of proteins in a functional unit (e.g. KEGG pathway map). Let *r* = |*G_c_*|,*k* = |*G*|,*z* = |*G_c_* ∩ *G*| and *l* the total number of proteins in the whole dataset. We assume that *z* follows a hypergeometric distribution. The probability to observe an intersection of size *z* between *G* and *G_c_* is computed as follows:
(5)
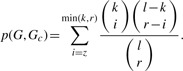

We then define the enrichment score *s*(*c*) of a component *c* by

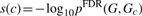

where *p*^FDR^(*G,G_c_*) is the corrected value of *p*(*G,G_c_*) by the false discovery rate (FDR) (Benjamini and Hochberg, 1995).

## 4 RESULTS

### 4.1 Extraction of canonical component sets of drug-targeted proteins and side effects

We applied the proposed SCCA method to the protein-binding profiles and side effect profiles (see ‘Materials’ section), which provided us with 80 canonical components. The correlated sets of proteins and side effects were extracted from each component. A list of drugs that contributed to the correlation was also obtained for each component. We refer to these correlated sets as canonical components (CCs) hereafter. All components present a limited number of proteins and side effects, which is a consequence of the sparsity of SCCA. This allows meaningful biological interpretation, indicating an advantage over OCCA.

**Fig. 1.**
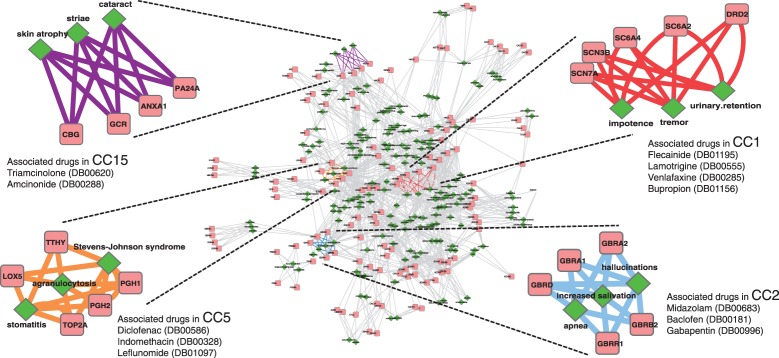
An illustration of the network of drug-targeted proteins and side effects in the extracted 80 CCs. Proteins (pink rectangles) and side effects (green diamonds) are connected if they appear in the same canonical component (CC). The highlighted CCs, 1 (red), 2 (light blue), 5 (orange) and 15 (purple) are discussed in Section 5. CC1: DRD2 (Dopamine D2 receptor), SC6A2 (Sodium-dependent noradrenaline transporter), SC6A4 (Sodium-dependent serotonin transporter), SCNs (Sodium channel protein subunits); CC2: GBRs (Gamma-aminobutyric acid receptor subunits); CC5: PGH1/2 (Prostaglandin G/H synthase 1/2), TOP2A (DNA topoisomerase 2-alpha), TTHY (Transthyretin), LOX5 (Arachidonate 5-lipoxygenase) and CC15: PA24A (Cytosolic phospholipase A2), ANXA1 (Annexin A1), GCR (Glucocorticoid receptor), CBG (Corticosteroid-binding globulin)

Figure 1 illustrates the network of extracted targeted proteins and side effects within the 80 CCs, where proteins (rectangles) and side effects (diamonds) are connected if they appear in the same component. The top five proteins and three side effects with highest weights are shown for easier visibility. The highlighted components CC1, CC2, CC5 and CC15 are further discussed in Section 5. The contents of all 80 CCs are listed in Supplementary Table S1.

### 4.2 Evaluation for canonical component sets based on targeted proteins

To evaluate the biological relevance of targeted proteins within the extracted 80 canonical components, we examine the functional units of proteins in two levels, biological pathways and molecular functions. Accordingly, we performed two kinds of enrichment analyses: (1) pathway enrichment analyses and (2) molecular function enrichment analyses. We used the KEGG database (Kanehisa *et al*., 2010) and the GO database (Ashburner *et al*., 2000) as gold standards for pathway and molecular function information. KEGG pathway maps and GO biological process terms were used in (1), whereas KEGG BRITE terms and GO molecular function terms were used in (2).

As summarized in Table 1, all 298 proteins extracted as members in the 80 canonical components were given molecular function annotations. 215 and 281 proteins were given pathway annotations by KEGG and GO, respectively. For each component, we computed an enrichment score for each of the functional units. A component was determined to be significantly enriched with a particular functional unit if the enrichment score, FDR-corrected *P*-value ≤0.05.

**Table 1.** Statistics in pathway and molecular function enrichment analyses

	(a)	(b)	(c)	(d)
Number of annotated proteins	215	281	298	298
Number of pathways/terms used in annotation	112	751	105	318
Number of components with enrichment	57	72	75	74
Number of enriched pathways/terms	33	93	50	75

Four types of functional units were tested; (a) KEGG pathway maps; (b) GO biological process; (c) KEGG BRITE terms and (d) GO molecular function.

**Fig. 2.**
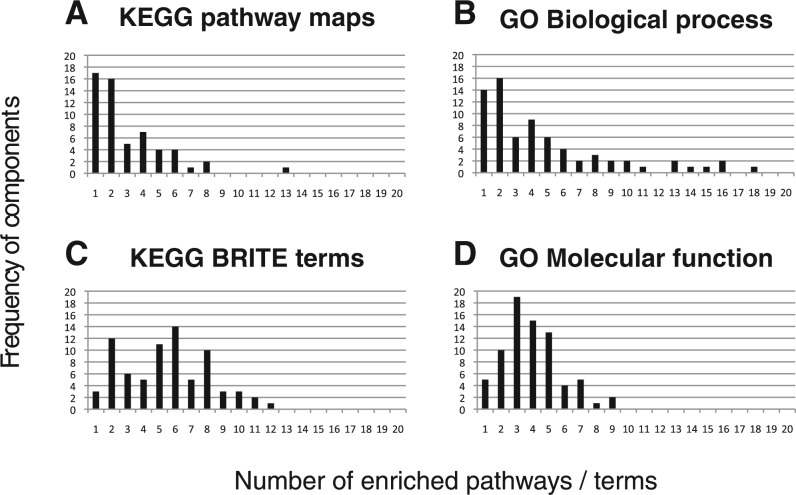
Canonical component distribution of the number of enriched pathways and molecular functions. For each of the 80 canonical components, enrichment score was computed in terms of the number of proteins associated with the component. The score was calculated for each of the functional units in two levels; biological pathways and molecular functions. Each histogram shows the frequency of canonical components against the number of enriched functional units associated with the components. **(a and b)** Pathway enrichment analysis using KEGG pathway maps showed that 33 components were enriched with one or two maps, and the other 23 components were enriched with <10 maps. For GO biological process terms, the frequency of components decreased as the number of enriched terms increased. **(c and d)** Molecular function enrichment analysis with KEGG BRITE terms showed much less skewed distribution compared to the distribution for KEGG pathway maps. GO molecular function terms showed a bell-shaped distribution with a mean at 3.95 terms. Comparison between the two enrichment analyses suggests that proteins extracted in a component are likely to be characterized by a limited number of biological pathways, even if their molecular functions are different

Figure 2 shows the distributions of canonical components against the number of enriched functional units associated with the component(s). The results of pathway enrichment analyses (Fig. 2a and b) displayed skewed distributions, i.e. the number of components decreased as the number of enriched pathways increased. Pathway enrichment analysis with KEGG pathway maps showed that 33 components were enriched with one or two pathway(s), and the additional 23 components were enriched with *<*10 pathways. This trend suggests that the majority of components were characterized by only a small number of KEGG pathways. Pathway enrichment analysis with GO biological process terms also displayed this trend. In contrast, the component distributions for molecular functions in Figure 2c and d showed much less skewness, and components were distributed across varying numbers of terms. This suggests that components are likely to represent a few distinct biological pathways for proteins from different molecular functions.

However, there remains a possibility that molecular functions of similar categories appear in a component. Thus, we also examined molecular functions of proteins in more general categories, such as ‘Ion channels’, ‘G Protein-coupled receptors’, and ‘Enzymes’. For the majority of components, as a result, proteins were annotated with more than one molecular function category, which confirms that proteins of very different molecular functions were grouped into the same component (Supplementary Fig. S1).

Table 2 shows the 10 most frequently appearing enriched KEGG pathway maps that showed enrichment. Component-based enriched pathways and associated enrichment scores are shown in Supplementary Table S2. Pathways co-appearing in the same component are biologically relevant. Among the 40 components that are enriched with more than one pathway, 14 contained two or three of the following pathways: ‘Calcium signaling pathway’, ‘Cardiac muscle contraction’, and ‘MAPK signaling pathway’. Interestingly, calcium is a signaling molecule that is well known to play an important role in muscle contraction, and there exists a direct cause-and-effect relationship between MAP kinase activation and smooth muscle contraction (Dessy *et al*., 1998). Therefore, in many cases where components contain more than one pathway, these pathways seem to take part in the same global biological function.

**Table 2.** Most frequently appearing enriched pathways

ID	KEGG pathway maps
map04080	Neuroactive ligand–receptor interaction
map04020	Calcium signaling pathway
map04728	Dopaminergic synapse
map04010	MAPK signaling pathway
map04260	Cardiac muscle contraction
map04727	GABAergic synapse
map04970	Salivary secretion
map04725	Cholinergic synapse
map00590	Arachidonic acid metabolism
map04270	Vascular smooth muscle contraction

All component-based enriched pathways and associated enrichment scores are shown in Supplementary Table S2.

We are aware that the KEGG pathways do not always describe signal transductions of proteins. For example, ‘Neuroactive ligand–receptor interaction pathway’ lists ligand–receptor interactions for G protein-coupled receptors (GPCRs) and ion channels. It describes protein families, rather than signal transductions occurring downstream of the ligand–receptor interactions. However, 18 out of 19 components were enriched with additional pathways.

These results indicate that component-based grouping of targeted proteins provides biologically relevant information in two ways. First, a significant number of proteins co-extracted in the same component are involved in the same biological pathway(s). Second, in many components, such proteins belong to different protein families. The component-based protein grouping cannot be inferred only from drug-targeted protein interactions, because they are often targeted by drugs of different chemical families. Accordingly, the side effects extracted in a component can be viewed as possible outcomes of biological pathway perturbations by drugs targeting the proteins that appear in the component.

### 4.3 Performance evaluation

It is difficult to evaluate the performance of the feature extraction method, because there is little knowledge about true association between targeted proteins and side effects. However, if the extracted components are biologically meaningful, they should contain some general properties which could be exploited for side effect prediction. We evaluate the performance of the method by recovering known drug side effect profiles from drug protein-binding profiles, using the extracted canonical components.

In previous literature, chemical structure fingerprints were used for predicting side effect profiles in the framework of OCCA (Atias and Sharan, 2011) and SCCA (Pauwels *et al*., 2011). Therefore, we made a comparison between chemical structure-based approach and targeted protein-based approach in the framework of both OCCA and SCCA by performing the following 5-fold cross-validation. First, drugs in the gold standard set were split into five subsets of roughly equal sizes, and each subset was used in turn as a test set. Second, the CCA model was trained on the remaining four sets. Third, the prediction score was computed from the test set, based on the components extracted from the training set. Finally, the model was evaluated for prediction accuracy over the 5-folds.

We evaluated the performance of the methods by the receiver-operating characteristic curve (ROC curve) and the Precision–Recall curve (Supplementary Fig. S2). The ROC curve is a plot of true positives as a function of false positives based on various prediction score thresholds, where true positives are correctly predicted side effects and false positives are incorrectly predicted side effects. The Precision–Recall curve is the plot of ‘precision’ (positive predictive value) as a function of ‘recall’ (sensitivity) based on various thresholds.

We summarized the performance by the area under the ROC curve (AUC) score and the area under the Precision–Recall curve (AUPR) score. To obtain robust results, we repeated the cross-validation experiment five times, and computed the mean and the standard deviation (SD) of the AUC scores over the five repetitions. Sparsity parameters *c*_1_, *c*_2_ ranged from 0 to 1 by 0.1 increments, and *m* ranged from 10 to 200 by 10 increments. The best results were obtained with *c*_1_ = 0.1, *c*_2_ = 0.1 and with *m* = 80 components in the case of SCCA. The same cross-validation experiments were repeated for OCCA (with no sparsity constraint), and the best results were obtained for *m* = 20 components.

Table 3 shows the resulting AUC and AUPR scores for the four different approaches, where the prediction scores for all side effects were merged, and a global ROC curve and a global PR curve were evaluated for each approach. This indicates that both SCCA and OCCA produce fairly good results and SCCA is slightly better than OCCA. It also seems that the targeted protein-based approach works better than the chemical structure-based approach. Results suggest that the targeted protein information is indeed useful for side effect prediction.

**Table 3.** Performance evaluation based on 5-fold cross-validation

Method	AUC ± S.D.	AUPR ± S.D.
Chemical structure-based approach		
Random	0.5000 ± 0.0000	0.0556 ± 0.0000
OCCA	0.8355 ± 0.0010	0.3753 ± 0.0016
SCCA	0.8708 ± 0.0007	0.3766 ± 0.0030
Targeted protein-based approach		
Random	0.5000 ± 0.0000	0.0556 ± 0.0000
OCCA	0.8850 ± 0.0007	0.4067 ± 0.0006
SCCA	**0.8895** ± **0.0002**	**0.4103** ± **0.0018**

Scores of the proposed method are highlighted in bold.

### 4.4 Prediction of side effects for uncharacterized drugs

In the DrugBank database, there are still 730 drugs whose target protein information is available, but side effects are not stored in the SIDER database. On the basis of their protein-binding profiles, we predicted the potential side effects for these uncharacterized drugs using the SCCA model, all of the 658 reference drugs being used as a training set. All prediction results can be found in Supplementary Table S3A. Complete analysis of all predictions is of course out of reach, so we focused on the side effect predictions of highest scores.

Some of the top-ranked predicted side effects involve Cinnarizine (DB00568), an anti-histaminic drug used against motion sickness. This drug binds to the histaminic H1 receptor, which is believed to explain its effectiveness in preventing vomiting in motion sickness. Its predicted ‘tremor’ (cyclical movement of a body part) side effect was confirmed by literature (Gimenez-Roldan and Mateo, 1991). Interestingly, Cinnarizine also binds to the voltage-dependent calcium channel involved in muscle contraction, which might explain the ‘tremor’ side effect. ‘Constipation’ is also predicted for Cinnarizine, as found in the adverse effect report 6127929-0 of the Food and Drug Administration (FDA). The predicted ‘dry mouth'side effect for Cinnazarine was also confirmed from literature (Gordon *et al*., 2001).

The second ranked predicted side effect is ‘diplopia’ (double vision) for Benzocaine (DB01086), a surface anesthetic that acts by preventing transmission of impulse along nerve fibers. Consistent with this activity, Benzocaine is an inhibitor of voltage-dependent sodium channel. The predicted side effect was confirmed from the literature (Horowitz *et al*., 2005). ‘Syncope’ was another side effect predicted for Benzocaine with a high score (Walker *et al*., 2003). The fourth ranked predicted side effect is ‘tremor’ for Bepridil (DB01244), an antihypertensive drug. Tremor is indeed one of the most common side effects for this drug, reported for 5% of all patients (Williams *et al*., 2002). ‘Tachycardia’ was also confirmed for Promazine (DB00420), an antipsychotic agent (Aronson, 2007). The side effect ‘diplopia’ for Nisoldipine (DB00401), a calcium channel blocker used for the management of hypertension, was also confirmed (O’Keefe and Creamer, 1987).

## 5 DISCUSSION

We provide biological interpretations of proteins and side effects extracted in each canonical component CC. Although CCs highlighted in Figure 1 are discussed here, the distinct characteristics were also observed in many other components.

**Fig. 3.**
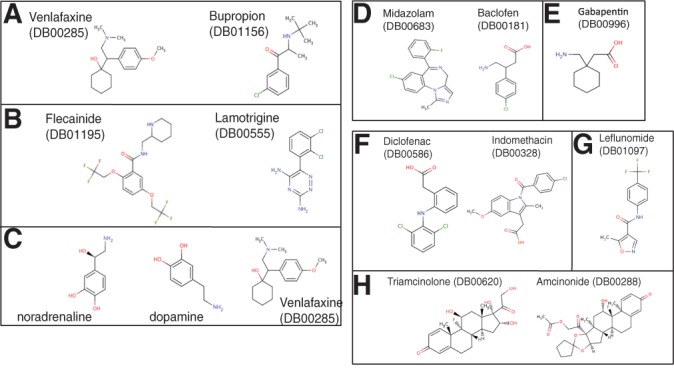
Examples of molecules responsible for extraction of CCs. **(a and c;** CC1) Venlafaxine and Bupropion bind to neurotransmitter transporters. Flecainide and Lamotrigine bind to sodium channels. Noradrenaline and dopamine are the two natural ligands of noradrenaline transporter and dopamine receptor, respectively. Venlafaxine also binds to noradrenaline and dopamine receptor. **(d and e;** CC2) Midazolam and Baclofen interact directly with GABA receptors. Gabapentin indirectly modulates GABA receptors activity by increasing GABA concentration in the synapse. **(f and g;** CC5) Examples of non steroidal anti-inflammatory drugs (NSAIDs) responsible for extraction of CC 5. They bind to prostaglandin G/H synthase (f) or arachidonate 5-lipoxygenase (g). **(h;** CC15) Example of steroidal anti-inflammatory drugs that bind proteins involved in the glucocorticoid signaling pathway. DrugBank IDs are provided in parentheses

In CC1, the top-ranked proteins were serotonin and noradrenaline transporters. They belong to the family of neurotransmitter transporters and are responsible for the release and re-uptake of the serotonin and noradrenaline neurotransmitter molecules by neurons, at the level of synapses. The uptake of the neurotransmitters is coupled to the co-transport of sodium ion by sodium channels to derive the required energy to pump the neurotransmitter against its gradient. Therefore, neurotransmitter transporters and sodium channels can be viewed as proteins playing part in the same biological process. This indicates that drugs-targeting sodium channels or neurotransmitter transporters share some side effects, indeed, drugs such as Venlafaxine or Bupropion that target neurotransmitter transporters gained high score in CC1, together with drugs such as Flecainide or Lamotrigine that target sodium channels. As shown in Figure 3a and b, channel blockers and drugs targeting serotonin and noradrenaline transporters display very diverse chemical structures, because they probably target very different binding sites. The fact that they lead to similar side effects could not have been foreseen based on analysis of chemical structures. Dopamine receptor was also found in CC1. This receptor belongs to the GPCR family and is implicated in many neurological processes such as motivation, cognition or fine motor control. Dopamine is a neurotransmitter that is structurally similar to noradrenaline, and in fact, it is a precursor of noradrenaline (Figure 3c). Consequently, a drug targeting one of these two proteins might also bind to the other, which might explain why these two proteins share some side effects and are found in the same component. Venlafaxine has a high score in CC1 and is an example of such a case.

In CC2, the top-ranked proteins were gamma-aminobutyric acid (GABA) receptors (GABA-A receptors). These receptors function as chloride channels gated by GABA. They mediate neuronal inhibition in the central nervous system. Consistent with this role, most of the high scoring drugs in CC2 were molecules that target GABA receptors and modulate their function. They are used as anti-anxiety agents, muscle relaxants or anesthesia adjuvants. Midazolam and Baclofen are examples of drugs targeting GABA receptors (Figure 3d), although these molecules belong to very different chemical families. Midazolam is a benzodiazepine molecule that binds to GABA-A receptors and acts as an agonist that increases GABA activity. Baclofen is a GABA derivative that targets GABA type B receptors (which are GPCRs) in addition to the GABA-A receptors. Interestingly, Gabapentin was one of the high scoring drugs in CC2 (Figure 3e), although it does not bind to GABA receptors. This molecule binds to and inhibits voltage-sensitive calcium channels. However, this drug is known to increase GABA concentration in the synapse, although the corresponding mechanism is not understood (Petroff *et al*., 2000). Consequently, it indirectly increases the activity of GABA receptors, which is consistent with its use (among others) as anti-anxiety agent.

Proteins extracted in CC5 and CC15 are mainly involved in pathways related to inflammation. In CC5, most proteins with high weights are enzymes belonging to the arachidonic acid metabolism pathway, such as prostaglandin G/H synthases, arachidonate 5-lipoxygenase and leukotriene A-4 hydrolase. Indeed, CC5 was enriched with the KEGG ‘Arachidonic acid metabolism’ pathway, in which proinflammation molecules such as leukotrienes or prostaglandins are synthesized. High scoring drugs in CC5 were mainly non-steroidal anti-inflammatory drugs (NSAIDs), that inhibit enzymes of this pathway. Some of these drugs are structurally unrelated, such as Diclofenac and Indomethacin, but they both inhibit prostaglandin G/H synthases (Figure 3f). Others also have very different chemical structures, such as Leflunomide that binds arachidonate 5-lipoxygenase (Figure 3g). However, all these drugs exert their anti-inflammatory action by inhibiting the same overall biological pathway, which explains why they lead to common side effects and contributed to the same component.

In CC15, most of the extracted proteins belong to the glucocorticoid signaling pathway, such as glucocorticoid receptor (a nuclear receptor) or corticosteroid-binding globulin. Glucocorticoids are a class of steroid hormones that are part of the feedback mechanism in the immune system that turns inflammation down. In this pathway, cytosolic glucocorticoid receptors are activated by glucocorticoid binding. The receptor–ligand complex translocates to the nucleus where it up-regulates anti-inflammatory proteins such as annexin 1, or down-regulates proinflammatory proteins such as interleukins or cytokines. High scoring drugs in CC15 were molecules from the steroid family such as Triamcinolone that binds glucocorticoid receptor or Amcinonide that binds glucocorticoid receptor and annexin 1 (Figure 3h). Therefore, although high scoring drugs in CC5 or CC15 present an anti-inflammation activity, they do not act on the same biological pathways. Consequently, they do not present the same side effects.

Interestingly, CC5 contained a human DNA topoisomerase. This protein controls the topological states of DNA, and therefore, one could wonder why this protein was extracted in CC5. In fact, various drugs from the fluoroquinolone family, namely, Ciprofloxacin, Ofloxacin and Doxorubicin, which target DNA topoisomerase were found among high scoring drugs in CC5. These fluoroquinolones and NSAIDs, or example Diclofenac and Indomethacin, share some of the extracted side effects of CC5, namely, ‘Stevens Johnson syndrome’, ‘stomatitis’ and ‘agranulocytosis’. NSAIDs and fluoroquinolone are structurally unrelated molecules, have completely different modes of action and target functionally unrelated proteins. The fact that they share the common side effects may not be explained by our current pathway data. Additional information such as relationships between pathways will be required to fully explain such cases.

Another case is a possibility that certain side effects can be caused by an alteration of the immune system introduced by drugs. It is known that synthetic glucocorticoids down-regulate the functions of immune cells (Flammer and Rogatsky, 2011), and certain anti-cancer drugs show immunosuppressive activities (Law, 2005). To discuss whether the occurrence of common side effects between these drugs is due to the direct inhibitions of the targeted proteins or indirect alterations of the immune system, an investigation of cross talks between signaling pathways and immune pathways is necessary.

## 6 CONCLUSION

In this article, we proposed a novel SCCA-based approach to relate drug targeted proteins with drug side effects. Using a cross-validation scheme, we found that the proposed approach displays better performance than chemical-structure-based methods for the prediction of drug side effects. Results suggest that side effect of drugs are more correlated to their mechanism of action, rather than to their chemical structure, which presents an interesting result. In most drug discovery projects, a therapeutic target playing a role in a given disease is searched for, and once identified, the corresponding pathways can be identified. The components that are enriched in these pathways provide a list of potential side effects that one can expect for future drugs acting on the target of interest.

We constructed a statistical model for the prediction of side effect profiles from protein-binding profiles, primarily because the number of drugs with side effect information is much less than those with targeted protein information. Indeed, it is unlikely in a practical situation that detailed side effect profiles are known for newly arriving drug candidate molecules. One limitation of our proposed method is that targeted protein information is not always obtainable; however, increasing information regarding protein-ligand interactions is becoming available from various biological assays. Thus, we envisage scenarios where a drug candidate molecules'f targeted protein information is available, but not side effect information. In this context, we believe that our proposed method presents itself as a powerful and informative tool for use within the drug discovery process.

*Funding*: (in part) JSPS International Training Program (ITP) and the Japan Science and Technology Agency. Computational resources were provided by Bioinformatics Center, Institute for Chemical Research, Kyoto University.

*Conflict of Interest:* none declared.
